# Dengue Prediction in Latin America Using Machine Learning and the One Health Perspective: A Literature Review

**DOI:** 10.3390/tropicalmed7100322

**Published:** 2022-10-21

**Authors:** Maritza Cabrera, Jason Leake, José Naranjo-Torres, Nereida Valero, Julio C. Cabrera, Alfonso J. Rodríguez-Morales

**Affiliations:** 1 Centro de Investigación de Estudios Avanzados del Maule (CIEAM), Universidad Católica del Maule, Talca 3480094, Chile; 2 Facultad Ciencias de la Salud, Universidad Católica del Maule, Talca 3480094, Chile; 3 Department of Engineering Design and Mathematics, Faculty of Environment and Technology, University of the West of England, Bristol BS16 1QY, UK; 4 Academic and ML Consulting Department, Global Consulting H&G, 8682 Sorrento Street, Orlando, FL 32819, USA; 5 Instituto de Investigaciones Clínicas Dr. Américo Negrette, Facultad de Medicina, Universidad del Zulia, Maracaibo 4001, Zulia, Venezuela; 6 Faculty of Engineering, Computing Engineering, Universidad Rafael Belloso Chacín, Maracaibo 4005, Zulia, Venezuela; 7 Grupo de Investigación Biomedicina, Faculty of Medicine, Fundación Universitaria Autónoma de las Américas, Pereira 660003, Colombia; 8 Master of Clinical Epidemiology and Biostatistics, Universidad Científica del Sur, Lima 156104, Peru; 9 Faculty of Medicine, Institución Universitaria Visión de las Américas, Pereira 660003, Colombia

**Keywords:** dengue, climate change, one health, machine learning, epidemiology, prediction, Latin America

## Abstract

Dengue fever is a serious and growing public health problem in Latin America and elsewhere, intensified by climate change and human mobility. This paper reviews the approaches to the epidemiological prediction of dengue fever using the One Health perspective, including an analysis of how Machine Learning techniques have been applied to it and focuses on the risk factors for dengue in Latin America to put the broader environmental considerations into a detailed understanding of the small-scale processes as they affect disease incidence. Determining that many factors can act as predictors for dengue outbreaks, a large-scale comparison of different predictors over larger geographic areas than those currently studied is lacking to determine which predictors are the most effective. In addition, it provides insight into techniques of Machine Learning used for future predictive models, as well as general workflow for Machine Learning projects of dengue fever.

## 1. Introduction

Dengue is a potentially life-threatening arboviral disease transmitted by female Aedes mosquitoes [1,2], especially *A. aegypti*, *A. albopictus*, and *A. vitattus*. These vectors are common tropical haematophagous ectoparasites. This zoonotic disease spread from African or Asian non-human primates 500 to 1000 years ago [3], but within the last 60 years it has spread from just 9 countries experiencing severe epidemics to become endemic in over 100 countries worldwide, even affecting non-tropical or subtropical areas [4,5]. Moreover, approximately one hundred million people yearly suffer from the symptomatic disease [4] caused by its four serotypes [6]. Given the significant impact of environmental changes on disease transmission, the One Health approach is urgently needed to implement the integration between human, animal, and ecological health.

The objective of this paper is to provide an insight into techniques that can be used for future predictive models based on the One Health perspective, particularly in respect to Latin America but also elsewhere.

One Health is a multidisciplinary approach that acknowledges the synergy between human and animal health and their shared environment. This idea is not new; the noted nineteenth-century pathologist (and originator of the term zoonosis) Rudolph Virchow famously asserted in 1858 that “between animal and human medicine, there are no dividing lines—nor should there be” [7,8].

This approach has become increasingly important in the 21st Century with the convergence of the pressures of changing climate, migration of human and animal populations, and the growing human population that increases the proximity between wildlife and humans. Indeed, the term One Health was only coined in the early 2000s with the appearance of the zoonotic SARS and H5N1 influenza diseases [9].

Whilst the One Health perspective is widely seen as necessary and increasingly used for better disease control [10], epidemiological approaches have not kept up with this change. Conventional epidemiological perspectives tend to view disease broadly from a human-only perspective, focusing on human demographic conditions with often only climatic/environmental factors accommodating the disease vector health. In contrast, One Health requires the health and lifecycle of the zoonotic disease vectors to be explicitly considered alongside the human environment, demographics, and interaction with the zoonotic host vectors.

For example, whilst environmental and sociological considerations often take a back seat in One Health [11], they frequently occupy the center stage in epidemiology [12]. Factors such as mean temperatures and rainfall used in predicting dengue [1], with a very vague consideration of how they affect the mosquito vectors, are an emergent challenge to be considered [13]. High rainfall, for instance, is beneficial to mosquitoes because it provides water-filled locations for eggs and larvae, whilst the mosquitoes are primarily impervious to strikes by raindrops that might otherwise kill them [14]. In addition, temperature and rain generally affect many other infectious and tropical diseases [15].

This article focusses on the assessment of the risk factors for dengue, with particular emphasis on South America, in an attempt to start to put the broader environmental considerations into a more detailed understanding and examination of the small-scale processes as they affect disease incidence.

In addition, the paper provides insight into techniques that can be used for future predictive models, particularly in Latin America and elsewhere. Techniques such as Machine Learning (ML) have experienced explosive growth that promises to revolutionize epidemiology and public health and offer new understandings of dengue and other infectious diseases.

The Elsevier, Emerald, Google Scholar, IEEE Xplorer, PubMed, ScienceDirect, Springer Science, and Taylor & Francis literature databases and search engines were searched for suitable papers in both English and Spanish, covering the full range of publication dates to 2022. Search keyword combinations included: dengue fever, prediction, machine learning, One Health, Latin America. The initial list of approximately 2000 papers was trimmed by eliminating multiple close article matches, personal opinions, and short articles (including conference papers, posters etc.).

## 2. Approach to Dengue Using the One Health Perspective

A recent epidemiological concern is the massive geographical expansion of dengue fever worldwide, which has led to a renewed interest in identifying critical determinants in disease transmission [16] that is part of a general trend over the past few decades. There has been a significant increase in the emergence and re-emergence of epizootics, zoonosis agents that can trigger epidemics worldwide [17]. The complex relationship between environment, human, and biological interactions is crucial to understanding the course of emergent infectious diseases and their implications for the public health sector [18]. In this respect, the One World-One Health initiative proposed in 2004 suggests a more holistic and transdisciplinary approach as a global strategy in the fight against those infectious diseases [17], particularly dengue fever in the Americas.

Traditional factors associated with dengue fever include social, economic, environmental, cultural, socioeconomic conditions, poverty, and factors related to the well-being of the inhabitants [1]. However, mosquito larval development and climatic conditions effects are some influential drivers that need further investigation [19]. Epidemiological experts claim that understanding the life cycles of relevant species in their natural habitats (in contrast to experimental conditions) could be a scientific challenge that must be overcome to move forward toward the construction of a more eco-inspired initiative for the control and prevention of emergent and re-emerged infectious diseases [17]. The density of infected agents, the diversity of the hosts, the viral replication, the migration rates of humans and other host species, and even the interaction between vector and host could guide us to elucidate more realistic scenarios to understand better the transmissibility of the diseases [16,17]. Considerations about human behaviors, in conjunction with vector population dynamics, are disease drivers strongly dependent on local ecology and social context [20]. Consequently, a more profound understanding of local environment is required. Although there is much discussion about the importance of climate change in the spatial distribution of infectious diseases worldwide [17,21], the lagged effects are also a local dependent characteristic [1] that needs to be better explained.

In addition, some authors have emphasized the importance of political agendas, economic inequalities, and cultural phenomena as factors that exacerbate the risk for the transmission of those infectious diseases in particular countries in Latin America [17,22].

In this respect, a systematic literature review undertaken by Hoyos W. [23] revealed a wide range of cofactors that have been recently included as newly identified drivers of dengue infections. This refined classification of predictors is presented in the following categories, all indicators of the One Health perspective. They have social (Soc), economic (Econ), demographic (Dem), population (Pop), environmental (Env), meteorological (Met), topographic (Top), climatic (Clim), dengue Baidu Search (DBSI), and Google Trends (G.T.) amongst others. The Dem, Soc, Econ, and Pop are measures that refer to the population features and typically include age, gender, type of housing, socioeconomic level, and others. The Clim, Env, Met, and Top refer to climatic and environmental changes, such as temperature, rainfall and altitude. Finally, the DBSI index, according to the study, refers to the data indicating increased interest amongst the general population, possibly due to increased community infections, identified by Internet search engines- for example, Google trends, Baidur Search Index and Twitter. These indicators are essential for dengue modeling and prediction [24,25,26,27]. Some other indicators are related to laboratory outcomes, referring to blood metabolites, such as leukocyte counts, hematocrit, albumin, and transaminase [23]. The considered clinical variables are the signs and symptoms in patients, including fever, blood pressure, joint pain, headaches, ocular pain, and migraine. There are also entomological factors for the vector insects, particularly, such measures as the Breteau index, container index, adult index, and predation rates, amongst others [23]. Even more, dengue, as other arboviral diseases [28,29], may lead to chronic and long-lasting clinical consequences [29,30,31]. The above studies considered genetic data, including gene expression, when available. In addition, some thermal imaging could also be valuable for prediction of patient morbidity [32]. Other measures mentioned by Hoyos [23] include cell phone data–referring to the data provided by mobile phones, such as geo-localization, and the mobility cofactors as drivers referring to passenger travel data, for example, destination country.

**Figure 1:**
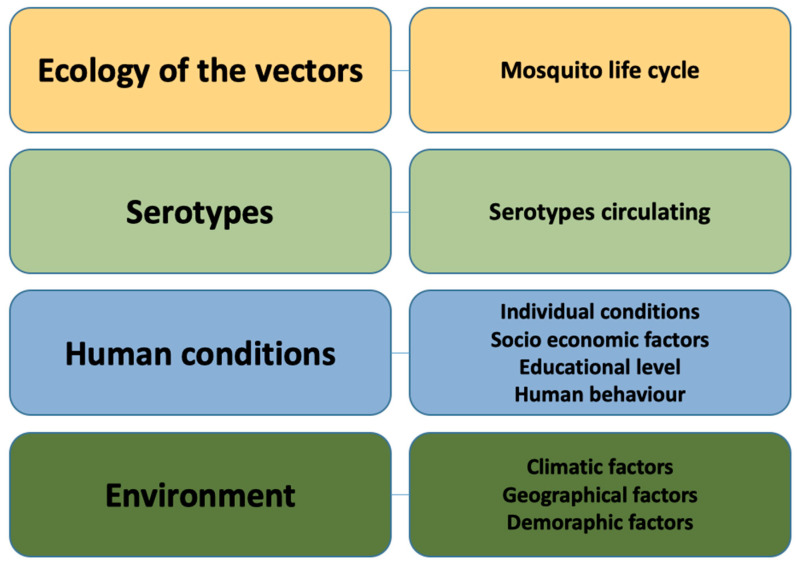
Risk factors of dengue fever are classified into four One Health groups: (i) ecology of the vectors, (ii) serotypes, (iii) human conditions, and (iv) environment.

As a result, this emerging understanding leads us to move toward a more holistic scenario of the Global Health perspective regarding dengue transmission. According to this perspective, we can classify the risk factors of dengue fever into four fundamental categories based on the One Health approach: (i) ecology of the vector, (ii) dengue serotypes, (iii) human conditions, and (iv) environment (Figure 1).

Although this review attempts to classify a wide range of factors focused on the One Health perspective, the geographical setting is relevant to gaining an understanding of the issues and limitations that exist within Latin America-focused studies of dengue fever, giving insights about potential challenges to be addressed, particularly regarding the availability of detailed information.

For example, a study conducted in Zulia state, Venezuela [1] suggests an urgent public health intervention to promote a national-scale social program to reduce the risk of dengue infection in Venezuela. That is a similar situation for Colombia and other countries in Latin America [33]. Furthermore, it emphasizes an urgent need for an efficient dengue fever surveillance network with explanatory variables at the finest spatial and temporal resolutions. We also encourage more innovative approaches to modeling dengue fever, such as using realistic scenarios to promote more effective interventions. That may be problematic in countries, such as Venezuela, where public health is neglected because of political crises [22].

Figure 1 presents a view of the One Health framework to show the following subcategories: (i) ecology of the vector (mosquito life cycle); (ii) dengue serotypes; (iii) human conditions (individual conditions, socioeconomic factors, educational level, human behavior), and (iv) environment (climatic factors, geographical factors, demographic factors). These will be used as guidance to help understand each cofactor’s implication in the presence and severity of the disease.

## 3. Ecology of the Dengue Vectors

Even though the *Aedes aegypti* mosquito is commonly found in tropical and subtropical areas, its distribution is modulated by environmental factors [34] that affect its life cycle, including lifespan, fecundity, survival rate, biting rate, disease transmission probability, infection probability, vector abundance, and incubation period [35]. As a result, *Aedes aegypti* is extremely synanthropic because of its anthropophilic nature and reproductive characteristics [34].

A good understanding of the dynamics of the disease using a One Health perspective requires a profound knowledge of the mosquito’s lifecycle. Therefore, it is necessary to consider the incubation periods in both mosquitos and humans and the biological cycles of the vector and virus [1]. For instance, a study in Thailand examined the complete mosquito life cycle, including egg laying, larval and pupal stages, and the adult period ranging from 7 to 12 days [36]. In addition, the virus circulating from an infected mosquito to a non-infected human host involves an intrinsic incubation period ranging from 3 to 14 days [37]. Subsequently, a viraemic period can manifest in humans with symptoms that last for six days, developing into the most severe stage of the disease [22].

According to these incubation figures, the maximum time for developing the most severe phase of the disease could be as long as 35 days. In the other direction, the virus can pass from an infected human to a non-infected mosquito with an extrinsic incubation period of between 8 and 12 days [37]. Some studies state that the maximum period from birth to adulthood of the mosquito is 12 days [36]. Consequently, with the full infection path in mind, the total period from the hatching of mosquito larvae to the final stage of the human disease is 59 days or approximately 2 months. However, variations are observed, which can be attributed to local weather conditions and environmental settings, which provide a non-homogeneous pattern regarding the spread of the disease in different parts of the world [38,39]. We will expand on such implications based on the environmental conditions.

## 4. Serotypes Circulating

Dengue fever is caused by an enveloped, positive single-stranded RNA virus of Flaviviridae with four different and genetically distinct serotypes DENV-1, DENV-2, DENV-3, and DENV-4 [40]. More recently, a fifth serotype has been found, although since it is sylvatic, it is currently of little consequence to humans [41]. A study performed in Vitoria Espirito Santo in Brazil between 2009 and 2013 [40] evaluated the relationship between the serotype circulating and the disease’s severity in this part of the world, and it revealed that DENV-2 caused seven times the incidence of severe illness than the other serotypes, consistent with similar studies conducted elsewhere [42,43]. Although the detailed mechanism responsible for the severity of DENV-2 remains unknown, it may be related to the high pathogenicity and rapid replication of this serotype, plus the stimulatory effect on the nitric oxide production considered responsible for the toxic and inflammatory effects in the host cells [40]. However, the authors also pointed to several limitations in the study, mainly because only 1.6% of the dengue cases in Vitoria Espirito Santo (Brazil) were serotyped.

Consequently, the early detection of dengue serotypes circulating in a particular area is crucial in controlling the number of severe cases of dengue. However, the authors also confirm the need to include factors related to serotypes and genotypes for further studies, with prospective approaches in hyperendemic settings and elsewhere related to susceptible hosts, such as demographic characteristics, co-morbidities and the immunological background of the hosts.

In addition, the study highlighted the recognition that multiple serotypes in multicenter settings can exacerbate dengue fever outbreak severity [44]. In this regard, potential secondary dengue infections and the sequencing of dengue genomes responsible for such diseases could contribute to understanding the complexities associated with dengue outcomes [40].

To identify the specific DENV serotype in a study performed from 2005 to 2010 in Peru, Bolivia, Ecuador, and Paraguay, serotype-specific monoclonal antibodies were generated using hybridomas [44]. In addition, the concentration of IgM and IgG immunoglobins was also measured, and the ratio of IgM/IgG was used as an indicator to discriminate between primary and secondary infection. However, this study’s main limitation was the extensive laboratory work needed to detect and identify the DENV serotype, which may be difficult in much of the Latin American setting.

The study also noted that the main symptoms of dengue fever could be classified into a set of groups: (i) constitutional symptoms such as malaise, headache, retro-orbital pain, and prostration; (ii) respiratory symptoms of cough, rhinorrhea, dyspnea, pharyngeal, congestion, cyanosis, rhonchi, and wheezing; (iii) gastrointestinal symptoms of diarrhea, nausea, vomiting, splenomegaly, hepatomegaly, abdominal distension, ascites; (iv) skeletomuscular symptoms, such as bone pain, myalgia and joint pain; (v) cutaneous symptoms including maculopapular rash, central erythema, distal erythema, facial erythema, vesicles, or subcutaneous nodules; and finally (vi) neurological symptoms of seizures, neck stiffness, impaired mental status, and focal neurological deficit. Crucially, the prevalence of the different clinical manifestations highly depends on the DENV serotypes involved.

## 5. Human Conditions

### 5.1. Individual Factors

Epidemiological studies of dengue fever conducted worldwide have provided evidence that age, gender, and race affect the severity of the disease [22,45,46,47]. For instance, a study conducted in Cuba revealed that Caucasians are less resistant than black African groups [46]. In addition, age has been closely examined as a risk factor in many studies performed in different parts of the world [22,45,47,48]. These confirm that children are the most vulnerable portion of the population for both mortality and morbidity of the disease.

In addition, gender has been identified as another important factor in many, but not all, epidemiological studies. For instance, some studies conducted in Latin America found no evidence of such gender-modulated susceptibility [22,49].

### 5.2. Socioeconomic Factors

Innovative approaches based on different methodologies have provided essential insights into how the urban environment or social network influences the well-being of the citizens in terms of dengue fever transmission [17] and how those findings might guide public health strategies in the fight against the disease.

A surveyed household study conducted in two urban locations within Machala in El Oro province in Ecuador found that the social factors associated with dengue fever might vary seasonally [20]. In addition, the mechanisms of seasonal influence on the host immune system may be affected by decreasing the coverage of artificial containers during rainy events [35]. However, further work is needed in this area.

Some investigations conducted in various parts of the world spotlight a strong association between the absence of public water supply and the severity of the disease [1,49,50,51,52,53,54]. Inadequate water supply coverage promotes using artificial containers to store water, consequently providing breeding sites for mosquitoes [55]. Such a lack of sanitary services might result from inefficient urban planning development [49,54,56].

In addition, some studies confirm that the low gross domestic product (GDP) per capita and higher population density [57] are potential indicators for dengue fever severity. Low per capita GDP can result in poverty and poor public health services. Other studies found that poor housing conditions and the percentage of empty houses and abandoned properties in the area are risk factors for the disease [19]. In this context, Ferreira and Schmidt [45] found that people who live in slums are more likely to become infected.

These conditions are often the result of inefficient governmental administration, in which unplanned urbanization, inadequate health infrastructure, ineffective disease control programs, and poor or no piped water and sewage services [20,49] are the main foundations for a marginalized society. For example, a recent study conducted in Zulia state, Venezuela [1] showed multiple socioeconomic risk factors for dengue, including the proportions of households without connections to a water supply network, homes without garbage collection services, families without properly installed showers, and those in homes surrounded by unoccupied dwellings. Other socioeconomic indicators are related to average household size and the number of people per room, mainly if the number of occupants per room is >4 [19]. Further socioeconomic risk factors include whether the head of the household is employed or, in contrast, if they are seeking work [19].

### 5.3. Educational Level

Several studies have demonstrated the relevance of promoting programs for the prevention of dengue fever, particularly in rural schools located in endemic locations in India, as a practical intervention for reducing exposure to the disease [58].

In this context, studies conducted in other parts of the world confirmed that the literacy rate is essential to the success of dengue preventive programs [59]. Moreover, indices such as head of household with post-secondary education and primary or lower educational level affect the severity of the disease in the home [19]. In line with the previous understanding of dengue severity, the mentioned factors are also crucial in avoiding the occurrence of the disease [20].

### 5.4. Human Behaviour

Common human behaviors also increase the risk of dengue outbreaks worldwide, including the carelessness in cleaning water containers used for washing and bathing [60]. These authors, working in India, found that most people leave water storage containers uncovered, inadvertently providing a good mosquito breeding site. Another factor related to human behavior is the presence of exposed rubbish surrounding the neighborhood. Discarded waste bottles, cans, plastic containers, and tires will likely become filled with rainwater, forming breeding sites for mosquitoes. Similar dengue outcomes based on measurements of rubbish surrounding the neighborhood have been confirmed in many other countries, such as Venezuela [53,59], Brazil [50,51], and Thailand [61].

In this context, the seasonal trends play an important role, not only because of the climatic and environmental factors but also because of their corresponding effect on human behaviors, including school schedules, holiday trips, travel patterns, week or weekend activities, and outdoor exposures [35].

Another crucial behavioral aspect is that *A. aegypti* are closely connected to human behavior because they enter dwellings to feed and rest. *A. aegypti* are mainly daytime biting species that can feed multiple hosts in a single gonotrophic cycle. In addition, the female mosquitoes can lay their eggs in artificial containers, including domestic decorative items, such as flower vases or pot plant bases [62].

Another issue regarding adult *A. aegypti* mosquitoes is the weakness of their intrinsic dispersal capability [62]. However, once eggs have been laid on the water surface, they can survive desiccation for up to a year, which allows the species an alternative mode of long-distance dispersal via human-mediated behavior, including intercontinental transportation on aircraft or ships [62].

## 6. Environmental Factors

### 6.1. Climatic Factors

Various studies have established the relationship between outbreaks of dengue fever and climatological factors, making dengue a climate-sensitive disease [1,37,38,52,63] in which temperature, rainfall, and humidity are significant drivers. In addition, the global phenomenon of El Nino Southern Oscillation (ENSO) aggravates the risk of dengue transmission in different parts of the world [16,38,52,64,65,66,67,68].

For example, a recent study conducted in Malaysia confirmed that rainfall, humidity, and wind speed are natural drivers that significantly affect the incidence of dengue in that country [69]. According to the authors, such findings could apply to many other subtropical regions.

Even though the seasonal pattern of dengue fever has been widely explained in terms of climatic factors, more studies are needed to better understand the short and long-term effects of those environmental conditions [35]. In addition, some experts suggest developing models around local or regional settings rather than more global models to increase the predictive sensitivity for dengue outbreaks [62].

Warmer temperatures affect the spread of the disease in various ways: firstly, a higher ambient temperature reduces the mosquito’s larva size, which produces smaller developed adults that digest blood faster [70,71]. Consequently, these adults require more blood to nourish their eggs [1]. Secondly, areas previously found to be inhospitable for developing a mosquito population became better, or even ideal, locations due to climate change [16,65,70,72]. For instance, dengue cases have recently been reported in uplands of Asian, African and Latin American regions, previously considered too cold for mosquito breeding [16,67,73], having now become places where mosquitoes thrive.

Some researchers have found that at certain key temperature levels, there is a reduction in the time it takes for the female mosquito to become infected after taking human blood (extrinsic incubation) [66,70]. Consequently, a faster dengue fever transmission rate is expected in those regions. For example, a study conducted in Mexico [70] reported that the average incubation period dropped to 7 days when the temperature was between 32 °C to 35 °C as compared to 12 days when the temperature remained at 30 °C.

In addition, rainfall events also favor the abundance of mosquitos due to the increase in potential juvenile habitats in the form of standing pools of water [20]. However, heavy rainfall can also destroy *Aedes aegypti* larvae [1,20,37]. Nevertheless, although heavy rain may reduce the number of individuals in the short term, it is more than compensated by increased breeding sites in the longer term [37]. Equally, low rainfall levels can lead to a rise in temperature, which unfortunately makes people more likely to use external water storage containers. This practice has increased dengue incidence in different parts of the world [1,20,60]. There is also a strong association between high humidity levels and dengue transmission [16,36,63], owing to the increased longevity of mosquitoes.

The inter-annual fluctuation of dengue fever outbreaks is strongly associated with the ENSO [65,74,75]. This cyclic phenomenon of ENSO, which originates in the Pacific Ocean, starts with oscillations in sea temperature from the east coast of Australia to the west coast of South America and causes global alterations in the atmospheric conditions every 2 to 7 years [72,75]. A recent study undertaken in Nepal revealed that dengue outbreaks in that country followed a cyclical pattern every 3 years, markedly during 2010, 2013, and 2016 [16]. Other studies have shown anomalies in temperature and rainfall associated with the El Niño phenomenon in some Latin American countries [64,75] and, consequently, severe outbreaks of the disease owing to the rise in mosquito population [72].

In this context, two extremes are associated with the ENSO phenomenon: El Nino (the warm event), characterized by the warming of the sea surface temperature and La Nina (the cold event). The impact of the ENSO on dengue transmission has been widely studied using a variety of indexes, such as the Sea Surface Temperature (SST) [52,64,70] in which the Pacific Ocean is split into five geographical zones capturing the mean sea surface temperature for each one, labelled: Nino1, Nino2, Nino3, Nino4, and Nino3.4 [64]. The Nino3.4 region is a significant overlapping zone between Nino3 and Nino4, dramatically impacting Latin American countries [1,52]. The outcomes resulting from [1] identified that the risk of El Nino3.4 fluctuates between 26.5 °C and 28.0 °C as a reference point for further comparations, also confirming the effect of the La Niña phase in the Zulia state, Venezuela.

### 6.2. Geographical Factors

The severity of dengue fever in some parts of the world is related to the length of the coastline, marked by the lowest altitude levels in conjunction with hot and dry weather conditions [53]. In this context, some investigations have found a strong association between low altitudes and the incidence of dengue fever [50,52,53,76]. One reason might be that biomechanical considerations suggest that *Aedes aegypti* species cannot fly in higher, thinner air [50]. Conversely, in another study, authors [1] found no evidence of variation of the disease in Zulia state, Venezuela, concerning altitude, suggesting that this measure might no longer be relevant to dengue transmission dynamics. Other studies have confirmed such findings because new outbreaks of dengue fever have been reported in regions without previous historical cases [16,77,78].

Other investigators have pointed to a correlation between the presence of the disease and rainforest due to the associated intense rainfall [66]. For example, a study performed in Peru during 1994–2004 revealed that dengue is highly persistent in the jungles for this reason [79]. Those findings suggested that the wilderness is an endemic area and triggers the expansion of dengue into the coastline. Therefore, to disrupt mosquito vector transmission, the best practice in coastal areas might be to focus mosquito destruction measures on adjacent jungle areas.

In addition, the considerable spatial heterogeneity of dengue fever in Guangzhou, China, as well as in other countries [13,21,57], suggested the importance of socioecological factors associated with urbanization, economy, accessibility, environment, and weather of which road density and water body locations proved to be dominant. According to these authors, water bodies exacerbated dengue transmission by providing mosquito breeding sites.

In this context, a recent Geographical index used in China [57] is the Normalized Difference Vegetation Index (NDVI), which may prove many uses. This index is computed from satellite imagery and takes a value between −1 to +1 [80]. If the value is negative, the image area is likely a body of water. An NDVI value close to +1 indicates a high possibility of having dense green leaves, and a value close to zero indicates an urbanized area.

### 6.3. Demographic Factors

Dengue fever is mainly an urban transmissible disease because it quickly spreads in urban contexts with a high human population density [51,52,53,81,82].

A mathematical modeling study revealed that commuting and day-to-day population movement are crucial drivers [83]. The spatial heterogeneity concerning the vector-host ratio and the travel patterns that people follow are critical components in dengue’s complex transmissibility, which has been recently supported by census surveys, cell phone use records, and GPS tracking techniques [83].

These authors emphasize the need for reliable information about human-vector contact in rural areas, which might be helpful in endemic settings in South America. Mathematical modeling simulated this via random movements that can be used for migratory exodus or regular commuter movements between patches representing residential or other locations [83]. However, according to these authors, the epidemic risk in a whole population comprises the contributions from the different demographic groups and their exposure levels to the mosquito population.

Another study performed in Queensland, Australia, found that areas with low GDP and high population density—usually peri-urban areas with poor hygiene conditions—promote a large local mosquito vector population and hence an increased incidence of dengue infection [57]. Hu et al., (2012) [84] agreed that whilst dengue fever is not naturally endemic in Australia, the *Aedes aegypti* mosquito has invaded northern Queensland, and the virus has been subsequently introduced to this local mosquito population by infected international travelers.

## 7. Machine Learning for Dengue Predictive Purposes in Latin America

### 7.1. Machine Learning Overview

The interest of the present work is a review of epidemiological predictive modeling based on ML techniques performed in the Latin American region. This section is devoted to presenting a short resume of ML concepts and, secondly, a review of applied ML studies of dengue fever, specifically predictive model applications to dengue fever outbreaks.

The recent growth in Big Data and the inclusion of Artificial Intelligence (AI) in different research fields have made these disciplines emerging scientific approaches. AI can be defined as computerized systems that can perform intelligent behavior [85]. Although AI covers a wide range of aspects and techniques, the most frequently mentioned are ML and Deep Learning (DL) [86]. Consequently, ML is a discipline of AI comprised of algorithms capable of finding patterns or learning from data, generating information for better decisions and predictions. On the other hand, DL is a subset of ML and is part of a family of methods based on Artificial Neural Networks (ANN), which are inspired by how the human brain works, learning from a large amount of data [87].

**Figure 2:**
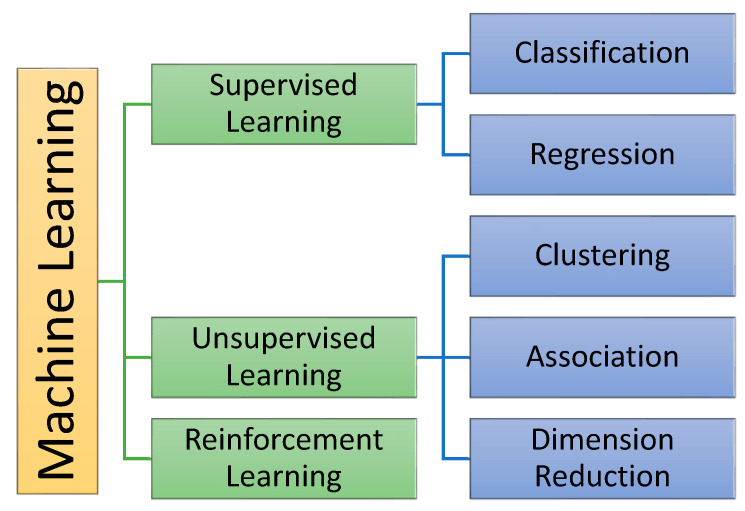
Types of learning are usually employed in Machine Learning.

ML uses three learning techniques; the first is Supervised Learning, which trains the model based on known inputs and outputs. Second, Unsupervised Learning is when the system tries to learn without previous knowledge and finds hidden patterns or intrinsic structures in the information. Finally, Reinforcement Learning or intelligent agents can observe the environment, take actions in the background, and get rewards in return [87]. Figure 2 shows a diagram of the types of learning usually employed in ML with corresponding applications.

**Figure 3:**
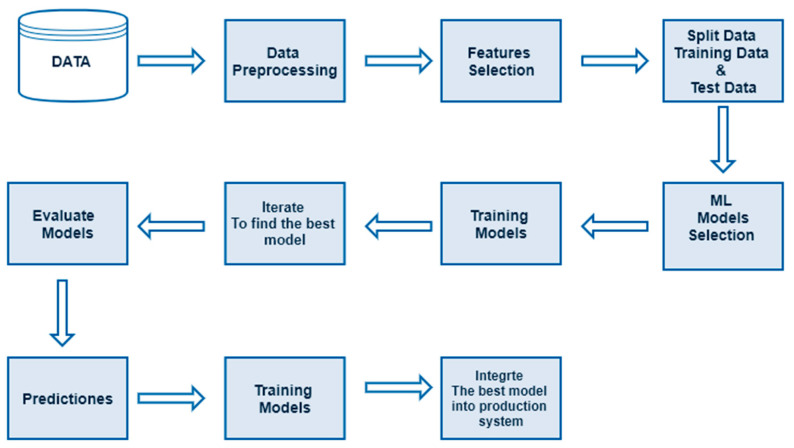
General workflow for ML projects.

In order to use ML, there are a variety of frameworks, which are interfaces, libraries, or tools that allow us to easily create ML models. Each of these frameworks is different from the others. Some of the best-known frameworks for ML are: (i) TensorFlow [88] is an open-source ML library developed by Google; (ii) PyTorch [89] it is an open-source and cloud platforms which is used by Facebook, IBM, among others; (iii) MatLab (MathWorks) [90] is a commercial programming and numeric computing platform that provides a variety of Toolbox for designing and implementing ML and DL models; (iv) Scikit-learn [91] is a free software machine learning library for the Python programming language. On the other hand, an ML project generally follows the workflow shown in Figure 3. 

Regression is one of the two fundamental tasks of supervised learning as shown the Figure 2. Therefore, two of the regression models introduce hereinafter:

Linear Regression Model [87,92]: A linear regression model predicts the target as a weighted sum of the feature inputs, formally:

The linear regression model assumes that the output variable *y* (a scalar) can be described as an affine combination of the *p* input variables $x_{1},x_{2},\ldots ,x_{p}$ plus a noise term *ε*,

(1) $$y=\beta_{0}+\beta_{1}\;x_{1}+\beta_{2}\;x_{2}+\ldots +\beta_{p}\;x_{p}+\varepsilon =\beta_{0}+\sum_{j=1}^{p}\beta_{j}x_{j}^{}+\varepsilon$$

where β_0_ is called the intercept, the β*_j_* represent the learned feature weights or coefficients. The term *ε* accounts for random errors in the data not captured by the model, i.e., the difference between the prediction and the actual outcome.

There are different methods to estimate the optimal weight. The ordinary least squares method is generally used to find the weights that minimize the squared differences between the actual and the estimated outcomes:

(2) $$\hat{\beta }=\arg\underset{\beta_{0},\ldots ,\beta_{p}}{\min}\overset{n}{\underset{i=1}{\sum }}{\left(y^{\left(i\right)}-\left(\beta_{0}+\overset{p}{\underset{j=1}{\sum }}\beta_{j}x_{j}^{\left(i\right)}\right)\right)}^{2}$$

The results of linear regression models are evaluated using a number of well-known metrics [87,92,93]. There are different models of linear regression, the major advantage of these models is linearity, it becomes the estimation procedure simple and easy to interpret. Due to this, linear models are consequently used extensively in fields such as medicine, sociology, psychology, and many other research fields [92].

**Figure 4:**
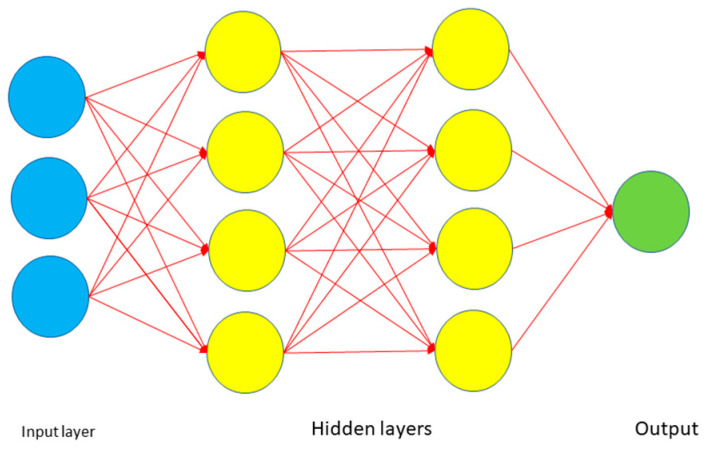
General architecture of an ANN.

Artificial Neural Network [85,94]: is an interconnected group of nodes similar to the vast network of neurons in a biological brain. They are a model inspired by the functioning of the human brain. The architecture of an ANN is shown in Figure 4 and consists of (i) the input layer that receives the input data and passes it to the first hidden layer; (ii) the hidden layers will perform mathematical computations with the inputs; and (iii) the output layer returns the prediction made.

**Figure 5:**
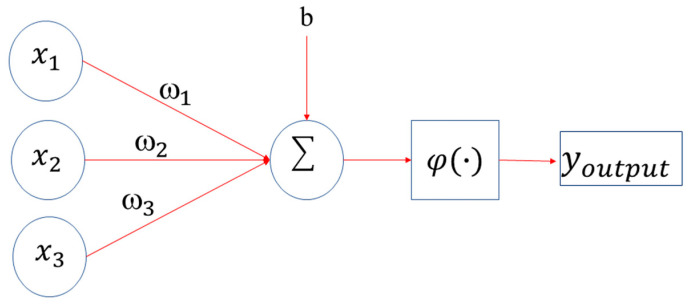
Illustration of the operations performed by each neuron in the ANN.

Each connection of the ANN neuron is associated with a weight. This weight dictates how important that relation will be in the neuron when multiplied by the input value (Figure 5). The output of the neuron is the weighted sum followed by the application of a nonlinear activation function:

(3) $$y=\varphi \left(b+\overset{p}{\underset{j=1}{\sum }}\omega_{j}^{}x_{j}^{}\right)$$

where *x_j_* are the input values in the neuron, *ω_j_* are the weights are adjusted, *b* is the bias and *ϕ*() is the nonlinear activation function, which will determine the behavior of the output of each neuron.

The ANN training process is based on calculating the error between the given labels and the output predictions using a loss function. Subsequently, the error is propagated through the network generally by means of the backpropagation algorithm updating the weights to minimize the error, repeating until converging or reaching a limit of iterations.

### 7.2. Machine Learning Applied to Dengue in Latin America

The use of ML to study dengue fever at different stages of the disease or for predictive purposes has emerged in recent years. A systematic review of studies that model dengue fever based on ML techniques [23] revealed three major research domains. These modeling approaches can address the diagnosis of the disease and its severity in which case the use of signs and symptoms is essential for developing the models. As a result, they are called models of prescription; secondly, the production of epidemic models, based on the analysis of the level of dengue severity within a selected population; in this domain, the models are used for predictive purposes; and finally, there are the models of intervention, consisting of the optimization and impact of the intervention programs [23], the authors create a list of the most common ML models for predictive purposes shown in Table 1.

However, a study [95] claims that no model for forecasting outbreaks of mosquito-borne diseases (MBD) could be adopted entirely, mainly because they cannot fit all the necessary conditions from the real world. According to the authors, the major constraint emerges due to the insufficient available data and the lack of open-source information via the internet, in conjunction with an unstructured and inadequate set of information, amongst other drawbacks. Therefore, the literature review of the MBD outbreak prediction framework in America [95] proposes an enhanced framework with the Entomological Index feature. In addition, ML is leveraged to increase the future MBD outbreak prediction.

Another complementary study centers on the global distribution of *Aedes aegypti* and *Aedes albopictus* as the main species responsible for dengue fever worldwide [96]. This study combined a high-dimensional multidisciplinary dataset with the ML techniques of Support Vector Machine (SVM), Gradient Boosting Machine (GBM), and Random Forest (RF).

There are some studies that focus specifically on ML techniques for dengue predictive purposes in the Americas; several studies compare the effectiveness of different predictive ML techniques. One of the more interesting is [97], which used a neural network model based on Long Short-Term Memory (LSTM) to predict future dengue cases. The predictors were collected weekly from 2016 to 2019, consisting of dengue incidence and the Egg Density Index from 397 ovitraps dispersed over the municipality of Natal, Brazil. Using dengue cases reported from previous weeks to forecast dengue incidence, the LSTM models and forecasting dengue incidence with ovitrap data showed a goodness-of-fit estimated by a correlation coefficient of 0.92 and 0.87, respectively. The results showed that ovitrap data allowed an earlier prediction of dengue outbreaks, of approximately 4 to 6 weeks, compared with just using the dengue itself, which was one week.

Furthermore, the authors in [98] implemented a recurrent neural network to forecast *Aedes aegypti* mosquito counts locally, using Earth Observation data inputs as proxies to environmental variables. The model was validated using in situ data from Vila Velha and Serra in Espírito Santo, Brazil and compared with RF and k-Nearest Neighbor (kNN) models, which showed a confidence adjustment of 95%.

Another study performed in Brazil [99] is based on dengue risk prediction across the whole country, using three types of ML algorithms with environmental and socioeconomic variables. Using monthly data from 2010 to 2013, the best performance was obtained with a RF approach.

In another study [100], the authors investigated the performance and viability of LSTM time series forecasting in predicting dengue cases compared to a Support Vector Regression (SVR) model based on a dengue dataset and satellite climate data comprising 936 weeks from 1990 to 2008 in San Juan, Puerto Rico. The findings revealed that LSTM showed superior performance and better-captured trends than SVR regarding the rise and fall of dengue cases. The performance of the models is evaluated using R^2^, mean absolute error (MAE), mean squared error (MSE), and root mean squared error (RMSE). Obtained for LSTM values R^2^ 0.075, MAE 8.76 MSE 245.61 and RMSE 15.67, compared to SVR results of R^2^ −0.13, MAE 18.02, MSE 1109.68, and RMSE 33.31.

Another study performed in the cities of San Juan (Puerto Rico) and Iquitos (Perú) [101] implemented a weather-related dataset to predict the number of cases per week using 13 different ML regression techniques. The dataset consisted of 1456 records and 24 features divided into five categories: location, temperature, precipitation, humidity, and vegetation index. The data was compiled in San Juan from 1999–2008 and the city of Iquitos from 2000–2010. The results show the Poisson Regression Model (PRM), Negative Binomial Regression model (NBM), and RF are the best with the lowest MAE of 25.6, 25.8 and 26.6, respectively.

In Mexico, some authors developed a holistic ML strategy for dengue fever using annual temperature [102] and a multi-stage combination of auto-encoding, window-based data representation and trend-based temporal clustering. The study used a trend association based on the Nearest Neighbor predictor. The epidemiological data corresponds to the number of dengue and dengue hemorrhagic cases collected from 1985 to 2010 within the 32 federal states of Mexico. The results showed the Autoencoding based Time Series Clustering with Nearest Neighbor achieves the lowest RMSE in 13 of 32 federal states but is the highest where another ML model is better.

ANN were used to predict dengue fever outbreaks in San Juan, Puerto Rico and the state of Yucatan, Mexico [103]. The data collected corresponded to 19 years of dengue cases in Puerto Rico and 6 years in Mexico. The study included environmental and demographic data such as sea surface temperature, precipitation, air temperature, humidity, dengue case epidemiological data, and population sizes. The authors applied two models in each area: one to predict incidence and the other to estimate vulnerable population size. They achieved a predictive power of better than 70%.

In another study [104], the authors used street-level images, such as those obtained from Google Street View, processed by Convolutional Neural Networks (CNN), to predict Dengue Fever (DF) and Dengue Hemorrhagic Fever (DHF) rates in urban locations in the city of Rio De Janeiro, Brazil, with data from 2010 to 2014. In addition, the authors evaluated simple and deep Siamese CNN architectures, comparing the results with both models. The study’s findings revealed a potential benefit in using deep CNNs and street-level images to identify DF/DHF hot spots in urban locations.

Finally, although the purpose of the present investigation was focused on ML applications in the field of dengue fever outbreaks, the holistic approach provides an open opportunity to learn from other vector-borne infectious diseases such as yellow fever [105]. Therefore, this study included a robust set of entomological data and landscape composition to predict areas permissive to yellow fever outbreaks. The measures for each sampling point were related to behavior, physiology, habitats, and epidemiological importance, as shown in Table 2.

## 8. Discussion and Conclusions

Many factors can act as predictors for dengue outbreaks. There are numerous small-scale studies that each examine a handful of these predictors in localized areas. However, although good results are obtained in these different small studies, there is an absence of a large-scale comparison of other predictors over larger geographical areas to indicate which predictors are the most effective.

There is little dengue epidemiological work yet that combines the One Health multidisciplinary response with ML techniques. Innovation in global health will need multidisciplinary work amongst ecologists, mathematicians, epidemiologists, and national and international government agencies for zoonic diseases using the One Health perspective.

AI is attractive because it offers powerful predictive capabilities for comparatively little effort and allows many disparate predictors to be easily incorporated into a model. However, some approaches, such as the novel use of street view image data by [98], cannot be done without ML techniques because of the difficulty in automatically selecting predictors from many features in an image.

AI is a promising approach to epidemiology because it can utilize many more factors than other approaches, whilst most mathematical and statistical epidemiological models typically only use a handful of predictors, ML approaches would have little difficulty coping with a much larger number of predictors. However, the major challenge with this approach is obtaining sufficient data to train the program, partly because data of the necessary resolution is difficult to find and, in many cases, is not even recorded.

For this reason, there is a strong need for a separate project independent of specific ML epidemiological approaches to obtain, collate, and publish in a suitable format likely predictor data for Latin America, ideally in a single publicly accessible repository on the web. That approach has been successfully used in many other areas of science, such as climatology, astronomy, and particle physics [94,106,107].

As previously stated, ML consists of a wide variety of models that can perform different tasks; the applicability of each model depends directly on the data and the task to be performed. Currently, the best-performing models are usually ensembles of several models [87,92]. There is no ML model that is better than another. This is evidenced in the study by Appice et al. [102] for predicting dengue outbreaks, where an ensemble of ML models showed the best results in 13 out of 32 states in Mexico, but this same ensemble obtained the worst results in the rest of the states. This is also the case for other studies predicting dengue outbreaks reviewed [100,103,104], showing good results for the modified RF model in some cases and in others with LSTM networks.

Whilst dengue fever has become endemic in many parts of the world, there are particular challenges in Latin America; whilst none are exclusive to this part of the world, the combination is.

## Figures and Tables

**Table 1:** Most common ML techniques for predictive purposes [23].

Abbreviation	Name of ML Technique
LoR	Logistic Regression
RF	Random Forest
LiR	Linear Regression
GAM	Generalized Additive Model
GLM	Generalized Linear Model
DT	Decision Trees
SVM	Support Vector Machines
ANN	Artificial Neural Networks
GBM	Gradient Boosting Machine
KNN	K-Nearest Neighbors
GWR	Geographical Weighted Regression
BRT	Boosted Regression Trees

**Table 2:** The measures related to behavior, physiology, habitats, and epidemiological importance of yellow fever can potentially be implemented in dengue outbreaks.

Mosquito Diversity	
Species richness	Total number of species sampled at each sampling point.
Shannon–Wiener index	Measure of species diversity weighted by the relative abundance.
Functional richness (FRic)	Represents the quantity of functional space filled by the community, where low FRic implies that some resources are unused or unavailable in the ecosystem.
Functional evenness (FEve)	Describes the distribution of abundance in a functional space of traits, where low FEve indicates that some parts of the functional niche are underutilized.
Functional divergence (FDiv)	A measure of the functional similarity among the dominant mosquito species of a community. FDiv is high when the most abundant species have extreme functional trait values.
Functional dispersion (FDis)	A multivariate measure of the dispersion of mosquito species in the trait space represents the mean distance of species to the centroid of the community, weighted by mosquito species abundance.
Haemagogus relative abundance	The number of Haemagogus mosquitoes is divided by the number of mosquitoes collected at each sampling point.
Haemagogus minimum infection rate (MIR)	Represents the minimum number of infected mosquitoes, assuming that only one was infected in each positive mosquito pool. It was calculated for each sampling point using the formula MIR = number of YFV-positive.
**Ecological Indexes**	
Environment of Mosquito sampling, inside the forest	Within dense forests connected to other forests.
Rural fragment	Within forests smaller than 100 hectares and surrounded by pastures.
Rural peri-domicile	Around homesteads and country houses.
Urban fragment	Within forests inside cities.
Urban intra-domicile	Within human houses inside cities.
Vertical distribution in the forest	
**Geo-Environmental Indexes**	
	Altitude, landcover/land use, forest fragment size.
	Normalized Difference Vegetation Index (NDVI).
**Functional Diversity**	
Physiology	Egg resistance to desiccation; larval development speed.
Habitats	Seasonal distribution; primary habitat.
Epidemiological importance	Epidemiological importance concerning the disease.
Behavior	Main hourly biting activity; host preference; oviposition preferences.

## Data Availability

Not applicable.
